# Cancer-work management: Hourly and salaried wage women’s experiences managing the cancer-work interface following new breast cancer diagnosis

**DOI:** 10.1371/journal.pone.0241795

**Published:** 2020-11-05

**Authors:** J. Kathleen Tracy, Fiyinfolu Adetunji, Gulam M. Al Kibria, Jennifer E. Swanberg

**Affiliations:** 1 Department of Epidemiology and Public Health, University of Maryland School of Medicine, Baltimore, Maryland, United States of America; 2 Department of Occupational Therapy, College of Health and Wellness, Johnson & Wales University, Providence, Rhode Island, United States of America; University of Technology Sydney, AUSTRALIA

## Abstract

**Objective:**

The purpose of this paper is to report the baseline characteristics of EMPOWER participants—a group of newly diagnosed breast cancer survivors—and describe differences in hourly and salaried wage women’s experiences regarding cancer and work management in the three months following breast cancer diagnosis.

**Design and setting:**

The EMployment and Potential Outcomes of Working through canceER (EMPOWER) project is a prospective longitudinal, mixed methods pilot study designed to evaluate how employment influences treatment decisions among women diagnosed with breast cancer. Participants were women diagnosed with new breast cancer and treated at one of two clinical sites of the University of Maryland Medical System. Women were enrolled in the study within three months of first breast cancer diagnosis. Study visits occurred every three months for one year. This paper reports data from for the baseline and three-month visit which had been completed by all enrollees.

**Methods:**

Trained research personnel collected demographic information, medical history and health status, social history, employment data, cancer-related data, psychosocial adjustment, and financial wellbeing at the baseline enrollment visit. A semi-structured qualitative interview was administered at the three-month study visit to assess employment decisions and the impact of job demands, cancer care, and cancer-work fit during the three months following diagnosis.

**Result:**

Fifty women with new, primary diagnosis of breast cancer were enrolled in the study. Mean age of participants was 51 years, and 46% identified their race as Black or other. The majority of women disclosed their diagnosis to their employer and nearly all maintained some level of employment during the first three to six months of treatment. Women with hourly wage jobs were similar to those with salaried wage jobs with respect to demographic and social characteristics. Women with hourly wage jobs were more likely to report working in physically demanding jobs and taking unpaid leave. They were also more likely to experience side effects that required physical restrictions at work, to leave their jobs due to demands of treatment, and to report managing cancer and work concurrently as very difficult. Women in salaried wage jobs were more likely to report falling behind or missing work and working remotely as a cancer-management strategy. Women in hourly jobs more often reported difficulty managing the competing demands of cancer and work.

**Conclusion:**

While further study is needed, these results suggest that women in hourly and salaried workers reported similar experiences managing cancer and work, with a few key exceptions. These exceptions pertain to the nature of hourly-wage work. Cancer survivors employed in hourly jobs may be more vulnerable to poor employment outcomes due to limited access to paid time off and workplace flexibility, and challenges related to managing physical aspects of cancer and employment.

## Introduction

Improvements in cancer screening and early detection in recent decades have increased 5-year survival across cancers by 70% [1] and led to increasing numbers of cancer survivors. Further, a majority (i.e., approximately 70%) of incident cancers are diagnosed in adults between the ages of 20 and 74 years old. Consequently, the vast majority of cancer survivors are employed at the time of cancer diagnosis and may continue to work and/or return to work following treatment. Identifying factors that allow cancer survivors to maintain employment or successfully return to employment is an important quality of life issue for cancer survivors and is also a significant public health issue.

Much of the extant literature has focused on return to work and factors related to cancer survivors’ ability to successfully return to work following diagnosis and treatment. In systematic reviews, Van Muijen and colleagues [2] and Mehnert et al [3] have noted treatment, employment, and demographic factors that are related to return to work. Specifically, chemotherapy and treatment side effects are negatively associated with return to work as are older age, low education, and low income. Employment characteristics such as workplace flexibility, job accommodations and supportive counseling or rehabilitation supports are positively associated with return to work for cancer survivors. What is less well-understood are the factors that foster or impede successful management of cancer treatment and employment ***while*** receiving treatment. Optimal management of cancer care and employment during active treatment may be influenced by features of the cancer care experience, employment needs and characteristics, or both.

**Fig 1:**
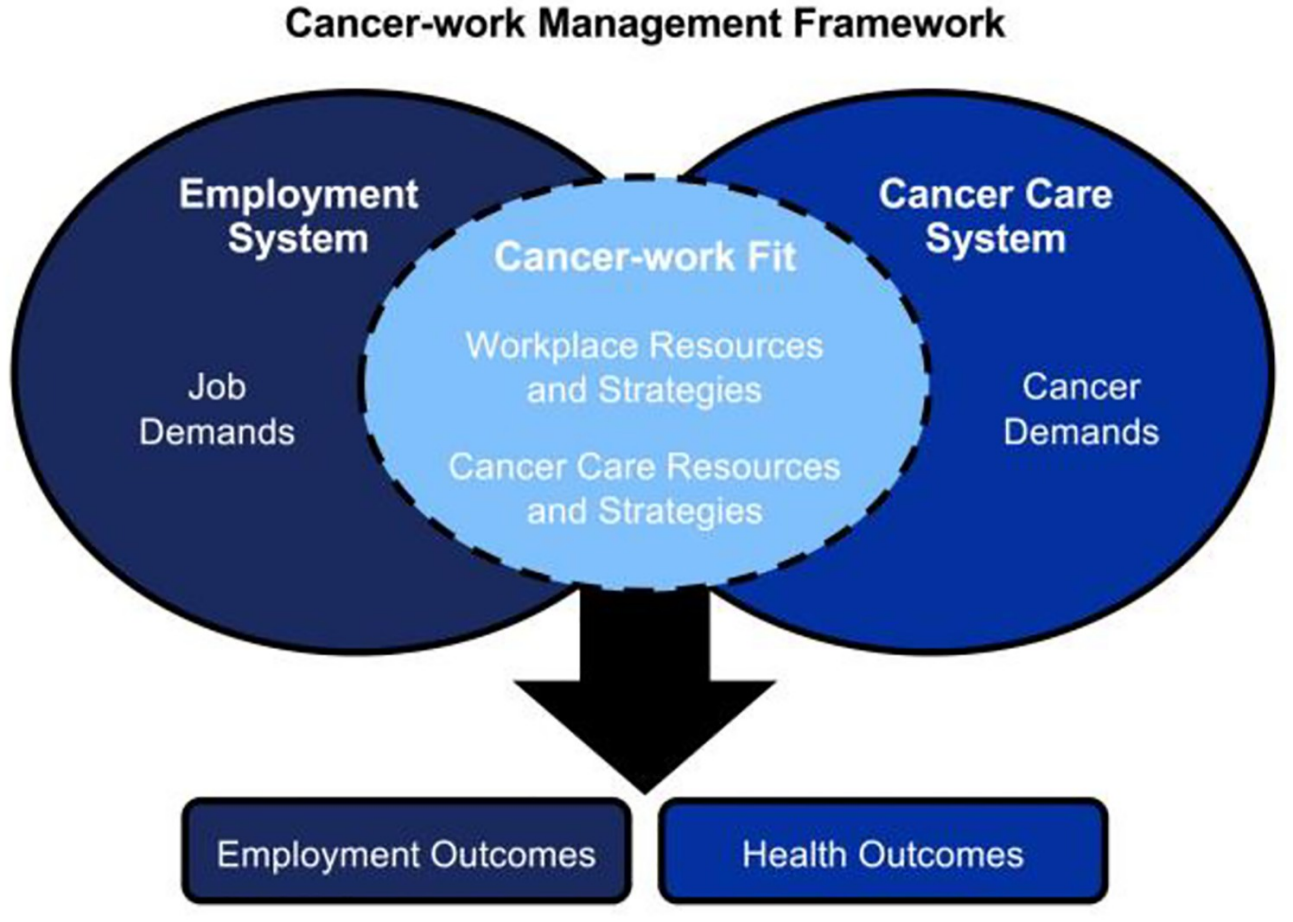
Cancer-work management framework. Source: Swanberg, Vanderpool, & Tracy (2020).

Swanberg et al, 2020 [4] recently offered the Cancer-work Management Framework as a conceptual model to explain the complex set of systems, resources, and strategies that influence employment and health outcomes for cancer survivors during active treatment (see Fig 1). Understanding survivors’ approaches to managing the potentially competing demands of cancer and work during active treatment and beyond requires recognition that a cancer survivor’s employment and cancer outcomes are influenced by both the cancer care and employment systems. Positive outcomes presumably reflect the extent to which the survivor is able to access resources and implement strategies to manage job and cancer care demands. Employment systems vary in complexity and characteristics. One major feature that is often correlated with other characteristics of the system is the type of compensation model under which an employee is paid—hourly vs. salaried compensation. In 2017, 58.3% of workers in the US (approximately 80.4 million individuals) were paid on an hourly basis [5]. Hourly wage jobs are often thought of as offering more limited access to employer-sponsored benefits, being more physically demanding, offering less autonomy and control, and having unpredictable and/or unstable work hours in comparison to salaried jobs. Despite the large proportion of workers in hourly jobs, relatively little is known about cancer survivors employed in these jobs, the impact hourly employment has on the lives of survivors, or whether their experiences differ from workers in salaried occupations. Consequently, it is important to explore whether there are differences in employment experiences of hourly and salaried cancer survivors and the impact of these differences. The EMployment and Potential Outcomes of Working through cancer (EMPOWER) Project seeks to address this important gap in our scientific and practical understanding of how breast cancer survivors manage cancer treatment and employment while actively receiving cancer care, especially during the first three months of treatment when women must adjust to the psychological implications of receiving a cancer diagnosis, while also adjusting their work and personal lives to accommodate cancer care. The purpose of this paper is to report the baseline characteristics of EMPOWER participants and to describe differences in hourly and salaried women’s experiences regarding cancer-work management. While women in the EMPOWER Project are followed for 12 months, this paper reports data for the baseline and three-month visit in an effort to better understand women’s choices immediately (i.e., within the first 90 days) following diagnosis.

## Methods

### Ethics statement

This study was reviewed and approved by the University of Maryland Baltimore Institutional Review Board (IRB), the University of Maryland’s Greenebaum Comprehensive Cancer Center’s Research Committee, and the Research Committee of the University of Maryland St. Joseph’s Medical Center. All participants provided written consent to participate in the study.

### Study design and sampling procedure

The data reported herein were collected as part of the EMployment and Potential Outcomes of Working through cancER project. The EMPOWER project is a longitudinal, mixed methods (qualitative and quantitative) pilot study designed to evaluate how employment influences treatment decisions among women diagnosed with breast cancer. A consecutive series of women with new breast cancer diagnosis (N = 50) were recruited from the University of Maryland’s Greenebaum Comprehensive Cancer Center (UMGCCC) and the University of Maryland St. Joseph’s Medical Center (UMSJMC) between July 2017 and October 2018. Women were eligible for participation if: 1) female; 2) 18 years or older; 3) they had been diagnosed with new, primary breast cancer within three months of enrollment (stages 0–4); 4) were employed at least 20 hours per week at the time of diagnosis; and 5) were able to read, speak, and understand English in order to provide informed consent and complete study procedures. Participation in the EMPOWER Study includes completion of study visits at five time points: baseline–which was within three months of being diagnosed with cancer, and then subsequently every three months for four additional visits. For the purpose of this study, we report results from the enrollment visit (visit 1) during which the interviewer assisted survey data were collected, and the qualitative interview conducted at visit 2. Women received compensation for completion of each study visit.

Potentially eligible women were identified through daily review of the electronic health records of UMGCCC and UMSJMC to identify women with new diagnosis of breast cancer. Diagnosis was confirmed with the respective cancer treatment team, and eligible women were sent a letter of invitation to participate in the study. Trained research staff contacted eligible women to explore interest in participation and to confirm employment status at the time of diagnosis. Women who were interested in participation were scheduled for an enrollment visit during which informed consent and completion of the baseline data collection occurred.

### Data and data collection

Trained research personnel collected demographic information, medical history and health status, social history, employment data, cancer-related data, psychosocial adjustment, and financial wellbeing at the baseline visit using an instrument comprised of measures described in more detail below. Quantitative data for the baseline survey were collected via a face-to-face interview-assisted survey method around the time of a medical appointment at the UMGCCC. Interviewers trained to conduct qualitative interviews conducted semi-structured qualitative interviews at the three-month visit to assess participants’ decisions related to continued employment, disclosure of cancer diagnosis to people at work, and workplace factors that influenced survivors’ employment decisions. Qualitative interviews were conducted over the telephone. No research personnel were involved in clinical care of participants.

#### Measures

*Demographics*. A structured demographics questionnaire collected information regarding age, race and ethnicity, sex, education, income, housing status, insurance data.

*Social history*. Social history data included marital/partner status, presence of children under the age of 18 living in the household, caregiver status, household size.

*Medical and health history*. Medical and health history data were collected from each participant’s electronic health record and by self-report. Medical and health history data included cancer diagnosis data (e.g., TNM staging, hormone positivity, treatment data) comorbidities, and tobacco and substance use history.

*Employment characteristics*. Self-report employment data included employment status, changes in employment, job characteristics (including type of work, size of employer, full-time v. part-time), length of employment, employment sector, and whether typical shift worked.

*Disclosure to employer*. Participants were asked a series of questions to determine the extent to which they had disclosed their breast cancer diagnosis at work. Questions addressed disclosure to co-workers, managers, and human resources personnel at the employer.

*Workplace supports*, *adjustments*, *and challenges*. Women were asked to provide information about resources (i.e., workplace supports) that were available to them through their employers to help them manage breast cancer diagnosis and treatment while continuing to work. Questions from the Medical Expenditures Panel Cancer Self-Administered Questionnaire (MEPS-CSAQ) [6] were used. Workplace support and adjustment questions included: paid time off, unpaid time off, changes to schedule (including reduction in hours, changes to scheduled hours or days), or changes to job duties or classification. Workplace challenges that were assessed included ability to keep pace with coworkers, productivity, and the ability to perform physical and/or mental job-related tasks.

*Barriers to cancer treatment*. Participants were asked to indicate the extent to which workplace and financial factors were barriers to treatment or affected treatment decision-making.

*Psychosocial adjustment*. Two broad domains of psychosocial adjustment were assessed: depression and stress. Symptoms of depression were assessed using the abbreviated Center for Epidemiologic Studies Depression Scale (CES-D 10 [7]). The CES-D 10 is a 10-item measure to evaluate symptoms of depression. Respondents rate the extent to which each statement has been true for her during the preceding week. Items are rated on a four-point scale (0 = rarely or none of the time; 3 = all the time), and items 5 and 8 are reverse scored. Total scores range from 0–30 with higher scores indicating greater symptoms of depression; scores greater or equal to 10 are considered reflective of depression.

Stress was assessed using the Perceived Stress Scale (PSS [8]) is a 10-item, Likert-type scale that requires respondents to rate the extent to which items have been true for the individual during the preceding month. Items are rated from 0 (never) to 4 (very often) and a total perceived stress score (range = 0 to 40) is derived by summing ratings across the 10 items. Individual scores are categorized as low stress (0–13), moderate stress (14–26), and high perceived stress (27–40).

*Financial distress/wellbeing*. Financial wellbeing was assessed using the InCharge Financial Distress and Financial Wellbeing (IFDFW [9]) scale is an 8-item Likert-type scale that is used to assess financial distress and financial wellbeing. Items are rated on a scale from 1–10 with higher scores indicating lower distress and higher wellbeing. A total score is derived by adding scores for each item and dividing by the number of questions. Total scores range from 1 to 10. Scores from 1–4 suggest high financial distress/low financial wellbeing; mean scores from 4.1–6.9 reflect average financial distress/financial wellbeing; and mean scores of 7–10 reflect low financial distress/high financial wellbeing.

*Semi-structured qualitative interview*. A semi-structured qualitative interview was conducted at the second study visit, which took place three months after the enrollment visit. Table 1 provides a summary of the topics explored during the qualitative interview. The Qualitative Interview was used to elicit information about women’s experiences related to employment context and each woman’s process for cancer-work management. Interviews were conducted, digitally recorded, transcribed verbatim, and coded by trained research personnel using Atlas.ti (Atlas.ti Scientific Software Development GmbH, Berlin, Germany).

**Table 1:** Topics explored by qualitative interview.

Topics
Background information
Employment overview
Job(s) held at time of diagnosis, including description of job tasks and responsibilities
Details of cancer-work management process
Information about cancer care demands that affected job responsibilities
If stopped working, cancer care and job-related factors that lead to that decision
Information about job responsibilities that influenced cancer-work management
Workplace environment
Job tasks and responsibilities; modifications required (schedule, tasks); challenges experienced at work during treatment; accommodations received from employer;
Disclosure of cancer diagnosis at work
Factors associated with disclosure of cancer diagnosis to co-workers, supervisors, others at work and responses to disclosure
Factors associated with co-worker and supervisor support of continued employment
Support from cancer care team
Exploration of supports/information/accommodations received from cancer care team to assist with cancer-work management and continued employment
Challenges associated with cancer care and continued employment
Cancer Treatment
Determination of side effects/aspects of cancer care and the effect of treatment on work ability
Influence of work on cancer care choices
Overall experience of cancer-work management

#### Analytic methods

*Quantitative data analyses*. Statistical analyses of quantitative data included descriptive statistics (frequencies or means/standard deviations [SD] to characterize the sample and scores on quantitative measures. Mean(SD) are presented for continuous variables and percentage(n) are presented for categorical variables. Fisher’s exact tests were used to examine differences on categorical variables, while Student’s t-tests were used to assess differences on continuous variables. Analyses were performed using Stata 15 SE (Stata Corp, College Station, TX). Given the pilot nature of this study and small group sizes, we report obtained p-values ≤0.10.

*Qualitative data analyses*. Qualitative data were transcribed and coded by trained research personnel to identify themes. Our approach to qualitative coding was a combination of framework analysis based on the Cancer-work Management Framework [4] followed by constant comparative method. Initial concepts from the Cancer-work Management Framework guided first round coding related to Job Demands, Cancer Demands, and Cancer-Work Fit: Workplace Resources and Strategies and Cancer-work Fit: Cancer Care Resources and Strategies. New codes were generated as the open coding process progressed. Nearly 250 codes were assigned as part of open coding, and definitions for each code were derived and entered into the Code Manager of Atlas.ti. Codes were reviewed and unified iteratively until there was 100% agreement between coders. For the purpose of this paper, codes relevant to the study objectives were aggregated into primary themes and sub-themes and reported accordingly. Crosstabulations were used to identify differences between hourly and salaried women on salient themes and sub-themes that emerged from the data. Qualitative coding and analysis were performed using Atlas.ti (Atlas.ti Scientific Software Development GmbH, Berlin, Germany).

## Results

### Participant characteristics

Table 2 provides a summary of participant characteristics at study enrollment for the full sample and for hourly and salaried wage workers. Fifty women were enrolled and completed the baseline visit. The majority of participants were diagnosed with Stage 0 or Stage I breast cancer, and all had insurance. The mean age of participants was 51(SD = 10.3) and 46% of participants identified as Black or other race/ethnicity. The majority had more than a high school education. Less than 50 percent of women were married; 37 percent were caregivers of children under 18 at the time of diagnosis, and 10 percent were caregivers to another adult. Nearly one-third reported significant symptoms of depression at enrollment and more than 50 percent reported moderate to high perceived stress. Forty-seven percent of women worked hourly jobs. Compared to women with salaried jobs, women who were hourly wage workers had significantly less education.

**Table 2:** Characteristics of EMPOWER participants at study enrollment/baseline (N = 50*).

Characteristic	%(n)	Hourly (n = 23)	Salaried (n = 26)	*P value*
Age [mean(SD)]	50.98 (10.3)	48.4(2.01)	52.8(2.03)	0.134
Breast Cancer Stage				0.74
Stage 0	18(9)	22(5)	15(4)	
Stage I	46(23)	44(10)	46(12)	
Stage II	26(13)	26(6)	27(7)	
Stage III	10(5)	9(2)	12(3)	
Race				0.47
Black	38(19)	48(11)	62(16)	
White	54(27)	48(11)	31(8)	
Other	8(4)	4(1)	8(2)	
Education				**0.02**
High school/GED†	12(6)	17(4)	8(2)	
Some college	24(12)	30(7)	15(4)	
2-year college degree	6(3)	4(1)	8(2)	
4-year college degree	28(14)	39(9)	19(5)	
Graduate degree	30(15)	9(2)	50(13)	
Marital status				0.99
Single/never married	30(15)	26(6)	31(8)	
Married	42(21)	43(10)	42(11)	
Separated	6(3)	4(1)	8(2)	
Divorced	16(8)	17(4)	15(4)	
Widowed	4(2)	4(1)	4(1)	
Lives with partner	2(1)	4(1)	—	
Caregiver for children < 18	37(18)	39 (9)	35(9)	0.02
Symptoms of Depression	33(16)	43(10)	23(6)	0.13
Moderate to high perceived stress	52(24)	45(10)	56(14)	0.47
Individual annual income [mean]	$69788.44	$60915.64	$78275.48	0.11
Income				**0.02**
Working poor	12(12)	61(14)	92(24)	
Working non-poor	6(38)	39(9)	8(2)	
More than 1 job (yes)	10(5)	9(2)	9(2)	1.00
Financial Distress/Wellbeing:				0.45
Mean (SD)	6.88(2.55)**			
High distress/low wellbeing	14(5)	21(3)	9(2)	
Average distress/wellbeing	32(12)	36(5)	27(6)	
Low distress/High wellbeing	54(20)	43(6)	64(14)	
Physical nature of employment				**0.01**
Mostly sitting	62(31)	43(10)	81(21)	
Mostly walking/moving	20(10)	26(6)	15(4)	
Walking and heavy lifting	18(9)	30(7)	4(1)	
Took unpaid time off (Yes); %(n)	26(13)	39(9)	12(3)	**0.04**
Difficult keeping pace at work (Yes); %(n)	39(19)	35(8)	42(11)	0.77
Decreased productivity (Yes); %(n)	37(18)	39(9)	35(9)	0.78
Treatment interfered w/ job-related physical tasks (Yes); %(n)	33(16)	48(11)	19(5)	0.07
Treatment interfered w/ job-related mental tasks(Yes); %(n)	35(17)	30(7)	38(10)	0.76

*N = 50 women recruited and completed the baseline visit. Some participants declined to answer all questions at the baseline visit, consequently the number with complete data for each variable varies slightly. Data related to comparisons of hourly v. salary wage workers pertains to 49 participants because one participant declined to share data that would allow us to determine her compensation model.

^†^GED: General Education Diploma;

**Financial distress measures were only available for (n = 37)

### Employment, income, and financial wellbeing

All women were employed at least 20 hours per week at the time of diagnosis; only six percent were unemployed by the time of their enrollment in the study. The majority of participants (94%) worked full-time. A small number of women (10%) had more than one job. Approximately a quarter of participants were categorized as working poor [following FY 2017 guidelines income limits provided by US Department of Housing and Urban Development] defined as those who reported incomes of 200% of the U.S. federal poverty guideline, and women who were hourly wage workers were significantly more likely to meet the definition for working poor. Approximately, 14 percent of women reported high financial distress in the period between enrollment and the 3-month visit; no differences in financial distress were noted between hourly and salaried wage workers. However, it is noteworthy that hourly wage workers were more likely than salaried workers to skip this question during the survey and that among those who answered it, 21% reported high financial distress, compared to 9% among salaried workers.

### Work disclosure, supports and adjustments, and workplace challenges

Nearly all participants (92%) disclosed their breast cancer diagnosis to someone (e.g., coworker, manager, human resources personnel) in the workplace, and no differences in disclosure were noted between hourly and salaried wage workers. With respect to workplace supports and adjustments (e.g., paid time off, unpaid time off, changes to schedule, changes to job duties or classification) a majority took either paid (44%) or unpaid (26%) time off for treatment and/or its side effects; hourly wage women were significantly more likely to have taken ***unpaid*** time off. Few women made changes to their job duties as a result of cancer diagnosis. In general, women rated overall quality of support from their employer for managing cancer-work challenges as good to high support.

A notable number of women reported that cancer treatment created workplace challenges that needed to be managed. Across all participants, 39% reported that cancer treatment made it difficult to keep pace with others/coworkers; 37% felt cancer treatment led to decreased work productivity; 33% felt treatment interfered with the ability to perform physical tasks required by their job; and 35% believed cancer treatment interfered with their ability to perform mental tasks required by their job. No differences were noted in the pattern of workplace challenges experienced by hourly wage workers compared to salaried workers.

### Barriers to treatment and cancer-work management

No participants reported deciding not to get recommended treatment due to cancer-work management conflicts; however, a small minority (8%) reported a change in the length of time (schedule) of treatment to accommodate work needs. Hourly wage women were more likely to report that they would have to make financial sacrifices as a result of cancer-work management issues (48% v. 23%, p = 0.08). Hourly wage workers were significantly more likely to be receiving financial assistance (p<0.01).

### Qualitative findings

Table 3 summarizes the job and cancer demands that women employed at cancer diagnosis encountered during the first three months of their cancer care. Job demands were those experiences associated with the physical requirements of the job; the perceived psychological strain experienced at work that was directly related to cancer care and treatment; and the perceived lack of workplace supports to assist them with managing their treatment. Perceived psychological strain associated with cancer-work management included women’s expressed concerns about managing co-workers’ and supervisors’ expectations of their work ability during active treatment, their own expectations about their work capability, or team members’ expectations about the speed at which they work. Perceived lack of workplace supports, included not having access to paid leave, workplace flexibility, or human resources related cancer support services. Cancer demands in this study are those cancer-related experiences that have a direct impact on work ability. Women described cancer demands as experiences associated with the nature of the cancer care, actions of treatment providers, cancer-related psychological distractions at work. Nature of cancer care included care demands that directly affected women’s work such as: the frequency of medical appointments, falling behind at work or missing work due to appointments and physical restrictions, and the side effects of treatment including physical restrictions and compromised immune system. Actions of treatment providers included timing of available medical appointments, lack of awareness of how cancer care influences women’s employment, and location of treatment facility to work or home.

**Table 3:** Job and cancer demands experienced by breast cancer survivors with hourly and salary jobs during the first 3 months of diagnosis by primary and secondary themes (n = 48).

**Perceived Job Demands**
Employed in a physical demanding job *
Perceived psychological strain at work due to cancer care
• worry about ability to continue standard workload (e.g. number of hours, tasks)
• concern about coworkers and/or supervisors' perceptions of ability to continue working during treatment
• lack of understanding or concern among coworkers about the radiating effects of a cancer diagnosis on work and personal responsibilities.
• continual management of own and work team's expectations work pace and productivity
Perceived lack of workplace supports to assist with cancer-work management
• no access to paid leave
• no access to workplace flexibility options*
• employer lacks programs or policies designed to assist cancer survivors with cancer-work management
**Perceived Cancer Care Demands**
Nature of cancer care
• frequency of required medical appointments
• fall behind or miss work due to frequency of appointments or physical restrictions due to treatment*
• side effects of treatment (physical restrictions* and compromised immune system)
Treatment Providers Practices
• medical provider only available for appointments during standard work day hours*
• perceived lack of awareness and concern of medical provider about implications of cancer on work ability
• proximity of treatment site from work or home
Cancer-related psychological distractions at work

*indicates that these experiences differed for women in hourly and salaried jobs. See Table 5 for more details.

Women implemented a number of cancer-work fit strategies as a way to incorporate cancer care into their work life routines. These cancer-work fit strategies were categorized into workplace strategies and cancer care strategies (see Table 4). Workplace strategies included workplace flexibility, taking short and long-term leave from work, resigning from work, making modifications at work (e.g. reduce job responsibilities, routine breaks, adjust work pace) and continuing to work without any required modifications. Cancer-care strategies were those modifications or adjustments made by a medical provider to accommodate her employment. These strategies included offering before-work appointments, relocating treatment to a site closer women’s home or work, and adjusting medical treatment in an effort to reduce side effects and maintain employment.

**Table 4:** Cancer-work fit strategies used by breast cancer survivors with hourly and salary jobs during the first three months of diagnosis by primary and secondary themes (n = 48).

**Cancer-work Fit Strategies: Workplace Strategies**
Workplace flexibility
• intermittent use of flexible scheduling of work hours (e.g. flex start/quit times)
• employer approval of modifying standard work schedule to accommodate treatment schedule
• work remotely from home to manage side effects of cancer treatment*
Work leave
• short term leave from work to manage side effects of treatment or attend medical treatment (e.g. take a day or two off intermittently)
• longer-term leave from work to cope with consequences of cancer treatment
Resign from Work*
Modifications at work
• reduce job responsibilities or take a demotion to a position with fewer job demands
• limits physical movements to avoid pain/strain at physician’s request or own volition
• take routine rest breaks to adjust to side effects of cancer treatment
• modify work pace to adjust to side effects of cancer treatment
No requested job changes to enable continued employment (e.g. continues working without disruption)
• fit required work hours around cancer care appointments
• time management allows for job task completion without having to work extra hours
**Cancer-work Fit Strategies: Cancer Care Strategies**
Modifications or adjustments made to cancer care by medical provider
• Offers early morning, pre-work, appointments in order to reduce job disruption
• Offers treatment at a location proximal to worksite or home
• Adjusts medical treatment to reduce side effects in an effort to enable continued employment

*indicates that these experiences differed for women in hourly and salaried jobs. See Table 5 for more details.

**Table 5:** Cancer-work management among workers with hourly and salaried jobs: A comparison of experiences (N = 48).

**Cancer-work Management Domains**	**Quotes from Workers with Hourly Jobs**	**Quotes from Workers with Salary Jobs**
Perceived Job Demands		
• Employed in a physical demanding job	"It’s constant up the steps, down the steps, up the steps, down the steps, lift him, lift her, lift him, lift her, get this wheelchair, get that wheelchair, put that leg on take that leg off, bathe this person and then at the end I’m like okay, now my legs are starting to hurt, and I don’t want to be at work and then my legs give out and I can’t take care of them."	“It’s a fairly active job, but it can be a desk job, too. There are days where I’m literally sitting you know, behind a computer doing IEP meetings all day, and then there’s other days where I’m um, out and about observing in classrooms or informally observing or running meetings or things like that.”
• NWF: no access to workplace flexibility options	“No.[there was no schedule flexibility] Because the lady that I was working for just was not with it. She just was like if you’re coming you’re coming, if you’re not you’re not.”	“I’m on contract and I have to work from 8:10 to 3:45. I can’t come in early, there’s no make-up time because I have to work when kids are there. If I wasn’t working I would be able to do it during the day, but um, you know I truly do feel I can work and it’s just my type of position working in a school doesn’t have a lot of flexibility in terms of taking time off because we have to be there around when the kids are there.”
Perceived Cancer Demands		
• NC: fall behind or miss work due to frequency of appointments or physical restrictions due to treatment	“I would say like pretty difficult [to manage cancer and work responsibilities.]…I Just trying to get everything done within the same time frame, and being gone most of the time, cause I didn’t extend my work day. I just had to do time management, that was about it. But nothing made it easier.”	“I have some kind of medical appointment, always twice a week, sometimes 3 times a week. So, I basically wound up working 4 to 5 hour days, but then I work from home in the evenings….I’ve missed a lot of meetings. It would be nice to have another 3 to 4 hours a day in the office and not missing meetings.”
• NC: side effects of treatment (physical restrictions)	“ah, I’m supposed to contact new accounts all the time. That was my main challenge. I wasn’t able to go to [visit] any new accounts. So I just stay connected with my old existing accounts which I have a good relationship with…but we constantly need to report how many new accounts we contact or develop.	“I wasn’t able to do anything due to the fact that I couldn’t move. I was in pain constantly. I told the surgeon, the oncologist, everyone, that this is worse than having 3 cesarean sections. So I did have pain but they don’t care, the only thing they say is go back to work to get your mind off things. They think just everybody works behind a desk.
• TPP: medical provider appointments available only during standard work day hours		“I’ve had a couple of appointments where I had to miss. My cancer doctor, his hours are a little more flexible; I can meet after school’s over. But my plastic surgeon, who did the reconstruction, she does not have those type of flexible hours. Meeting with her has been hard. I have an appointment this Friday, so I’ll miss a couple hours of work because of that.”
Cancer-work Management Domains	Quotes from Workers with Hourly Jobs	Quotes from Workers with Salary Jobs
Cancer-work Fit Strategies: Workplace Strategies		
• WF: work remotely from home to manage side effects of cancer treatment	“I took the day [I had surgery] off ha, um, and then because um, like, I couldn’t shower and I was all bandaged up, my job allowed me to work from home for the next week, and then the week after that, and I had some doctor’s appointments so you know, I just took leave during the time I had doctor’s appointments. The following week I had radiation treatment. It was twice a day for five days with six hours between treatments. My supervisor said you don't have to work, take the time to take care of yourself. I chose to work a few hours each day in between my treatments. “	“So, I have a really good team that works for me. And they were able to pitch in. They would continue to email me…. on days when I wasn’t so tired I would read my emails from home and [I would] get caught up. When I came back [to work], my business manager gave me the run-down on everything…When I took another week off after radiation, I worked from home. Since I wasn’t the one initiating contact with external parties, that’s kind of how I managed i, I was letting my team be the face and I would just help them behind the scenes.”
Resign from Work	“the only influence [i had to stop working] was the fact that my immune system was going to drop and gyms aren’t exactly the cleanest place. I couldn’t really be around that many people when I have no immune system. [I left my job] about two weeks after I found out, because as soon as I got the diagnosis, within I’d say eight days I was starting treatment. I gave them [my employer] my two weeks notice so I worked through the weekend of my first chemo treatment but that was it.”	None

Note: Abbreviations were used to illustrate the primary theme in which the experience was affiliated. Concepts without abbreviations are primary themes. NWF: No workplace flexibility; NC: Nature of cancer care; TPP: Treatment provider practices: WF: Workplace flexibility.

**Fig 2:**
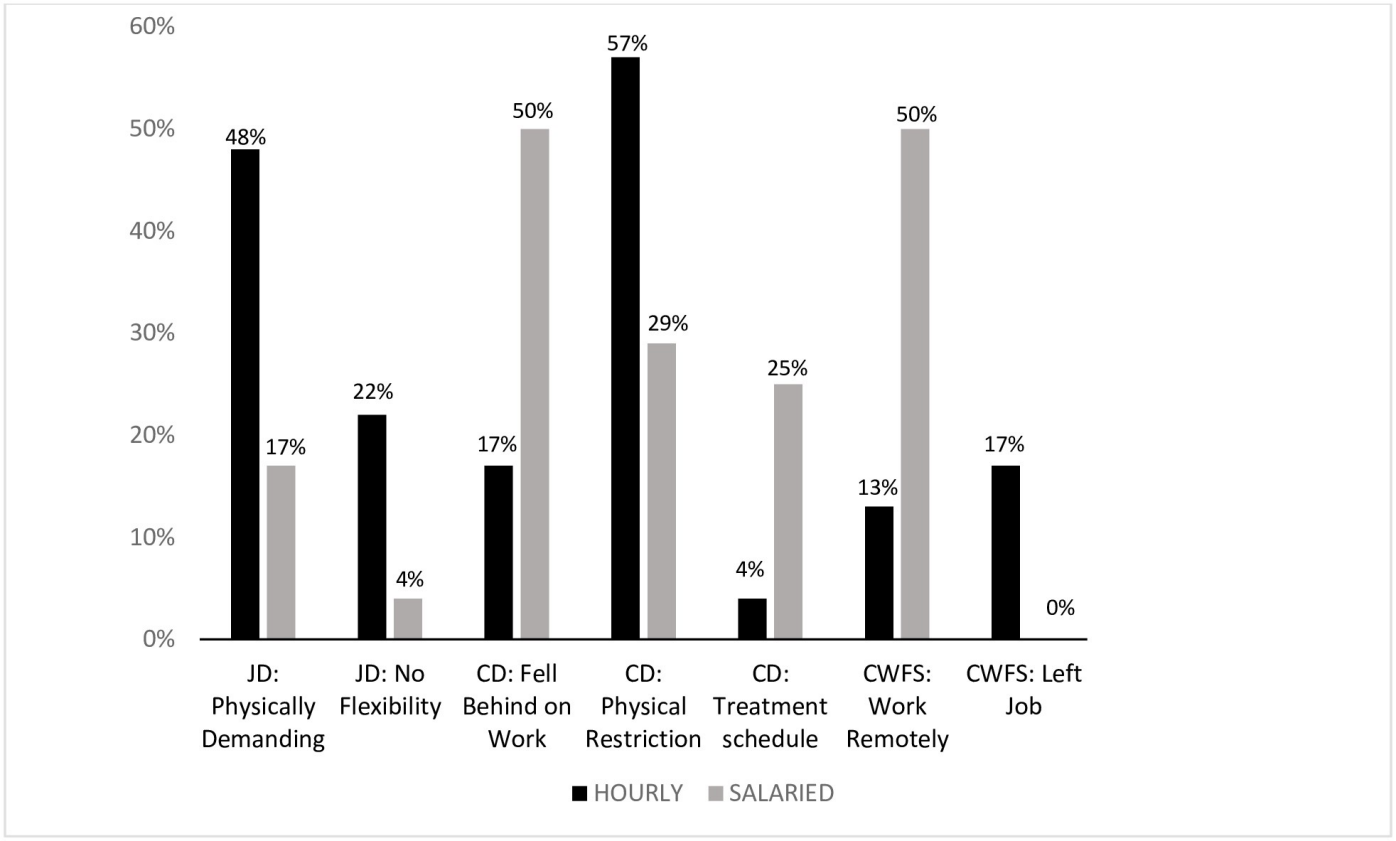
Comparison of hourly and salaried wage breast cancer survivors on themes related to cancer-work management (N = 48). Notes: CD: Cancer care demands; JD: Job demands; CWFS: Cancer-Work Fit Strategy.

Women with hourly and salary jobs had several different cancer-work management experiences (see Fig 2). With respect to perceived job demands, women with hourly jobs were more likely than those in salary jobs to discuss physical demands of their jobs (p = 0.03) and not having access to workplace flexibility (p<0.10). Falling behind at or missing work due to the frequency of medical appointments or required physical restrictions was a cancer demand more commonly reported among women with salaried jobs (p = 0.03) as was the lack of availability of medical appointments other than during the standard work day (p < .10). Women with hourly jobs were more likely to report needing to make physical restrictions at work due to side effects of cancer treatment(p = 0.06). While, women with hourly jobs were more likely to leave their job (p = 0.05) as a cancer-work fit workplace strategy, women with salaried jobs were more likely to access working from home as a approach to maintain employment during active treatment (p = 0.01). Women with hourly jobs were more likely than women in salaried jobs to report managing cancer-work-fit as very difficult when answering a specific question during the qualitative interview that sought to assess their level of difficulty managing cancer care and work concurrently (p = 0.05).

## Discussion

The current study is one of the first the authors are aware of to characterize breast cancer survivors’ cancer-work management during active cancer treatment. The objective of this paper is to report the baseline characteristics of EMPOWER participants and to describe differences in hourly and salaried women’s choices regarding cancer-work management during the three months following new breast cancer diagnosis. During the first three months of treatment women must abruptly adjust to the psychological implications of receiving a cancer diagnosis, while also adjusting their work, personal and family lives to accommodate cancer care. Nearly all survivors enrolled in this study maintained employment during the first three to six months of treatment. Similar to what is observed for the general employed population [10], women with hourly jobs reported less education and were more likely to also be categorized as working-poor. Hourly wage survivors were otherwise socially and demographically similar to women with salaried wage jobs.

Nearly all participants disclosed their breast cancer diagnosis to their employer and/or coworkers. Seventy percent of participants took time off work for treatment or because of treatment side effects; 26% took unpaid time off, and survivors in hourly wage positions were more likely to take unpaid time off than their salaried wage counterparts. Women with hourly jobs were also somewhat more likely to report that cancer management interfered with their ability to perform physical aspects of their jobs. Both hourly and salaried wage breast cancer survivors generally felt they had access to adequate workplace supports and accommodations and that their workplace supported the need for flexibility of schedules in order to manage cancer and work. It is noteworthy, however, that women with hourly jobs were significantly more likely to need to make physical accommodations on their jobs in response to cancer care demands, to leave their jobs altogether due to the demands of treatment, and to express that managing cancer-work fit was very difficult. This is in contrast to women in salaried jobs who were more likely to share that they were able to work remotely as a strategy for maintaining employment during active treatment.

Results from the qualitative portion of this study illuminate aspects of the Cancer-work Management Framework [4] and provide insight into women’s experiences managing cancer and work concurrently during the initial months of new breast cancer diagnosis. While job demands created different challenges for women with hourly jobs, all women expressed concerns about managing their own as well as others’ expectations about their capacity to fulfill their job responsibilities. A general lack of understanding about breast cancer and breast cancer treatment, in combination with a lack of awareness about how the progression of treatment would influence women’s workability over the course of treatment seemed to weigh heavily on women’s mind. The lack of access to workplace supports in many cases compounded concerns about cancer-work management. Workplace policies related to privacy and confidentiality of health information may create challenges for employers to more proactively support employees as they undergo medical treatment, and in our case, cancer care. However, given the desire—and necessity for some—survivors to continue employment during treatment, this is an important area for future research.

The cancer care delivery system was another important factor in women’s strategies for cancer-work management. The timing and frequency of appointments, as well as the proximity of the cancer care center contributed to women’s decision to continue to work, whether they needed to take time off and for how long, and ultimately if they would need to take extended leave or resign from their job. Providers often communicate directly and indirectly that treatment should be prioritized above all else; in reality, cancer survivors have rich lives filled with competing joys and responsibilities that create complex cancer-management scenarios that are seldom as simple as “treatment comes first.” Cancer care providers’ awareness and concern about women’s jobs also played a role in many of the participants cancer-management experiences. Offering early morning appointments or appointment times that did not conflict with women’s work schedules eased concerns about missing work or needing to take leave. Similarly, switching to care locations proximal to home or work allowed women to minimize their time away from work thus easing the ability to remain employed while receiving cancer care.

Our study operationalizes job and cancer demands experienced by working breast cancer survivors during active treatment. and the strategies used to manage the cancer-work interface. In reality it is the intersection of all of these experienced demands and readily accessible resources and supports available to women at work and through their treatment team that influence women’s decisions about how they navigate treatment while working. Given that a majority of our participants wanted or needed to continue working while receiving cancer care, cancer care teams should consider ways in which they can engage collaboratively with survivors to develop treatment decisions and plans in ways that support continued employment if that is a priority for the survivor.

While cancer-work management is challenging for any cancer survivor, results from this study suggest the occupational conditions inherent to hourly jobs may influence women to make decisions that could influence financial stress and well-being, long-term employment, and job security. The longitudinal design of the EMPOWER study will allow researchers to examine the associations between job conditions and outcomes over time.

### Limitations

The current study offers new insights related to how hourly and salaried wage women manage employment decisions following new breast cancer diagnosis. Even so, our study had several limitations that should be acknowledged. First, categorizing occupations into binary categories does not take into account variation in job conditions that may exist within the hourly and salary categories. However, we opted to use these categories because we wanted to understand the experiences of workers in jobs that frequently have few employer-based resources with those workers who may be more likely to have employer-sponsored resources to assist with cancer-work management. Hourly work is a term that is synonymous with low-wage work, and salaried jobs are typically thought of as jobs that come with standard employee benefits and are these job classifications are frequently used by other researchers [11, 12]. Second, the sample size recruited for this pilot study was relatively modest. As a result, our findings should be considered preliminary and should be replicated. Our study also focused on women receiving cancer care from a large academic health system, a care setting that likely doesn’t reflect the cancer care experience of most patients with breast cancer. Findings should be replicated with larger samples and should explore populations of women that are receiving primary breast cancer care from community hospitals rather than a major academic health system. The resources of an academic health center are quite different from those available to community-based hospitals, especially academic health systems that also have NCI-designated Comprehensive Cancer Centers affiliated with their systems. It is possible that the challenges hourly wage women face compared to salaried women in managing cancer and employment may be greater in community settings where supportive resources available from the cancer care system are less robust. A final limitation of the current study was the small overall number of women that made significant employment changes in order to meet demands of managing cancer and work. The relatively small number that discontinued employment precluded our ability to identify factors most strongly associated with changes in employment among women with new breast cancer diagnosis.

### Summary and future directions

Hourly wage women in our study were somewhat more likely than salaried wage women to report challenges managing cancer and work. This included needing to take more unpaid time off in order to meet the demands of treatment, having lack of flexible work arrangements, needing to make modifications (i.e., restrictions) to physical aspects of their jobs, and reporting that managing work and cancer treatment was very difficult. Hourly wage women were also more likely than their salaried counterparts to leave their jobs altogether due to the demands of cancer treatment. Our study is the first we are aware of to examine differences in cancer-work management by job type–hourly vs salary. The differences that emerge indicate that at least at the beginning of treatment, the types of workplace supports that are beneficial differs between the two groups. Because hourly wage women were more likely to take unpaid time off and leave their job altogether, cancer treatment may put this group at a higher risk of financial stress over the course of treatment.

Future research is needed to better understand the influence of occupational conditions on employment and cancer decision making of breast cancer survivors, financial stress and well-being, and employment outcomes. This study sets the foundation for future multi-variate analysis to examine direct and in-direct effect of job conditions on these stated outcomes. Data from the EMPOWER study will allow for this type of exploration and possibly form the basis for a larger study of cancer-work management among breast cancer patients in hourly and salary jobs. Likewise, understanding the interplay between the physical demands of cancer treatment, job conditions, and other factors that influence cancer-work management (e.g. provider supports) could help to identify key areas for interventions that might ease challenges experienced by survivors managing cancer and work.

## References

[1] Siegel R, Miller K, Jemal A. Cancer Statistics, 2019. *CA CancerJClin*. 2019;69(1):7–34. 10.3322/caac.21551 30620402

[2] Muijen PV, Weevers N, Snels I, et al Predictors of return to work and employment in cancer survivors: A systematic review. *Eur J Cancer Care (Engl)*. 2013;22:144–160. 10.1111/ecc.12033 23279195

[3] Mehnert A, de Boer A, Feuerstein M. Employment challenges for cancer survivors. *Cancer*. 2013;119 Suppl 11:2151–2159. 10.1002/cncr.28067 23695927

[4] Swanberg JE, Vanderpool RC, Tracy JK. Cancer–work management during active treatment: towards a conceptual framework. *Cancer Causes Control*. 2020;31(5):463–472. 10.1007/s10552-020-01285-1 32125547 PMC13551477

[5] Characteristics of minimum wage workers, 2018: BLS Reports: U.S. Bureau of Labor Statistics. Accessed July 26, 2019. https://www.bls.gov/opub/reports/minimum-wage/2018/home.htm

[6] Institute NC. Medical Expenditure Panel Survey (MEPS): Experiences with Cancer Survivorship Supplement.; 2011:992–1004. http://healthservices.cancer.gov/surveys/meps/10.1007/s11764-012-0221-2 PMC6362834 23011572

[7] Björgvinsson T, Kertz SJ, Bigda-Peyton JS, McCoy KL, Aderka IM. Psychometric Properties of the CES-D-10 in a Psychiatric Sample. *Assessment*. 2013;20(4):429–436. 10.1177/1073191113481998 23513010

[8] Cohen S, Kamarck T, Mermelstein R. A global measure of perceived stress. *J Health Soc Behav*. 1983;24(4):385–396. 6668417

[9] Prawitz AD, Sorhaindo B, Kim J. The Incharge Financial Distress/Financial Well-Being Scale: Establishing Validity and Reliability. Published online 2006:13.

[10] SHRM_Profile_of_the_Hourly_Worker.pdf. Accessed September 29, 2019. https://redeapp.com/wp-content/uploads/2016/02/SHRM_Profile_of_the_Hourly_Worker.pdf

[11] Kalleberg AL. *Good Jobs*, *Bad Jobs*: *The Rise of Polarized and Precarious Employment Systems in the United States*, *1970s-2000s*. Russell Sage Foundation; 2013.

[12] Vanderpool RC, Swanberg JE, Chambers MD. A Narrative Review of the Confluence of Breast Cancer and Low-wage Employment and Its Impact on Receipt of Guideline-recommended Treatment. *Glob Adv Health Med Improv Healthc Outcomes Worldw*. 2013;2(5):75–85. 10.7453/gahmj.2013.046 24416698 PMC3833560

